# Activity of two hyaluronan preparations on primary human oral fibroblasts

**DOI:** 10.1111/jre.12602

**Published:** 2018-09-27

**Authors:** Maria B. Asparuhova, Deniz Kiryak, Meizi Eliezer, Deyan Mihov, Anton Sculean

**Affiliations:** ^1^ Laboratory of Oral Cell Biology School of Dental Medicine University of Bern Bern Switzerland; ^2^ Department of Periodontology School of Dental Medicine University of Bern Bern Switzerland; ^3^ Biozentrum University of Basel Basel Switzerland

**Keywords:** gene expression, growth factors, hyaluronic acid, oral soft tissue wound healing, pro‐inflammatory cytokines

## Abstract

**Background and Objective:**

The potential benefit of using hyaluronan (HA) in reconstructive periodontal surgery is still a matter of debate. The aim of the present study was to evaluate the effects of two HA formulations on human oral fibroblasts involved in soft tissue wound healing/regeneration.

**Material and Methods:**

Metabolic, proliferative and migratory abilities of primary human palatal and gingival fibroblasts were examined upon HA treatment. To uncover the mechanisms whereby HA influences cellular behavior, wound healing‐related gene expression and activation of signaling kinases were analyzed by qRT‐PCR and immunoblotting, respectively.

**Results:**

The investigated HA formulations maintained the viability of oral fibroblasts and increased their proliferative and migratory abilities. They enhanced expression of genes encoding type III collagen and transforming growth factor‐β3, characteristic of scarless wound healing. The HAs upregulated the expression of genes encoding pro‐proliferative, pro‐migratory, and pro‐inflammatory factors, with only a moderate effect on the latter in gingival fibroblasts. In palatal but not gingival fibroblasts, an indirect effect of HA on the expression of matrix metalloproteinases 2 and 3 was detected, potentially exerted through induction of pro‐inflammatory cytokines. Finally, our data pointed on Akt, Erk1/2 and p38 as the signaling molecules whereby the HAs exert their effects on oral fibroblasts.

**Conclusion:**

Both investigated HA formulations are biocompatible and enhance the proliferative, migratory and wound healing properties of cell types involved in soft tissue wound healing following regenerative periodontal surgery. Our data further suggest that in gingival tissues, the HAs are not likely to impair the healing process by prolonging inflammation or causing excessive MMP expression at the repair site.

## 1 INTRODUCTION

Hyaluronan (HA) is a naturally occurring non‐sulfated glycosaminoglycan involved in maintaining extracellular matrix (ECM) resilience and tissue hydration. HA is present in various body fluids such as synovial fluid, serum, saliva, and gingival crevicular fluid^1^, ^2^, ^3^ as well as in mineralized and non‐mineralized tissues, including the periodontium.^4^ Higher amounts of HA are detected in gingiva and periodontal ligament^5^ than in cementum^6^ and alveolar bone.^7^ Due to its hygroscopic and viscoelastic properties as well as high conservation among species, HA has been utilized in a number of biomedical applications in dermatology, ophthalmology, osteoarthritis treatment, oral and maxillofacial surgery as well as in various tissue engineering applications.^8^ Although HA is involved in many different biological processes during tissue repair and regeneration, detailed mechanisms of action especially in oral soft tissue wound healing following periodontal regenerative procedures remain largely uncovered.

During wound healing, HA exhibits differential effects based on its molecular weight (MW).^9^ In early stages, there is a sharp increase in high MW (>1000 kDa) HA, which has the ability to bind fibrinogen, a reaction intrinsic to clot formation.^10^ The initial large HA polymer is anti‐angiogenic and immunosuppressive, facilitating polymorphonuclear leukocyte access to the wound site for removal of dead tissue, debris and bacteria. Thereafter, in the inflammatory stage, HA fragments of lower MW (<700 kDa) accumulate due to hyaluronidase activity or oxidation.^11^ These are able to induce production of pro‐inflammatory cytokines such as tumor necrosis factor‐α (TNF‐α), interleukin‐1β (IL‐1β) and IL‐8^12^ as well as angiogenesis.^13^ In periodontal wound healing in particular, HA has been shown to induce production of pro‐inflammatory cytokines by fibroblasts, keratinocytes, cementoblasts, and osteoblasts, which promotes the inflammatory response and consequently stimulates hyaluronan synthesis by endothelial cells.^14^

The wound healing process involves a number of events rigorously controlled by matrix metalloproteinases (MMPs) and growth factors including transforming growth factor‐β1 (TGF‐β1), platelet‐derived growth factor (PDGF), fibroblast growth factor‐2 (FGF‐2), and epidermal growth factor (EGF).^15^ MMPs degrade ECM components and elicit a pro‐inflammatory response, thus promoting cell migration during wound remodeling.^16^ PDGF induces cellular responses throughout all phases of the repair process.^17^ TGF‐β1 has been recognized as a key regulator of collagen expression.^18^ FGF‐2 plays a role in re‐epithelialization, angiogenesis, and granulation tissue formation but also contributes to matrix synthesis and remodeling, which are critical for the wound healing process.^19^ Similarly, EGF is a potent stimulator of epithelialization, angiogenesis, fibroblast proliferation, and survival.^20^

A number of studies describe the use of exogenous HA in non‐surgical and surgical periodontal therapy with generally beneficial but moderate effects on surrogate outcome variables of periodontal inflammation, ie, bleeding on probing and residual pocket depth.^21^, ^22^ However, only few studies exist on the use of HA in reconstructive periodontal surgery.^23^, ^24^, ^25^, ^26^ Before such clinical studies are conducted, a better understanding of the influence of HA on the behavior of oral fibroblasts involved in periodontal regeneration is needed. Thus, the goal of the present study was to investigate the in vitro effects of two commercially available HA preparations of non‐animal origin planned to be used in reconstructive periodontal surgery. The influence of the two HA preparations on the proliferative and migratory abilities of primary human palatal and gingival fibroblasts, as the main cell types involved in soft tissue regeneration in the oral cavity, was investigated. Furthermore, we studied the rapid activation of signaling proteins elicited by the two HA formulations. We hypothesized that HA stimulates the wound healing potential of oral fibroblasts in vitro and would thus contribute to soft tissue healing/regeneration following reconstructive periodontal surgery.

## 2 MATERIAL AND METHODS

### 2.1 Cell culture and HA preparations

Primary human palatal (HPF) and gingival (HGF) fibroblasts were obtained from three donors each using tissue explant technique. Tissue samples harvested from either subepithelial palatal connective tissue grafts (for obtaining HPFs) or subepithelial buccal gingival tissues (for obtaining HGFs) were retrieved from systemically and periodontally healthy anonymous individuals below 30 years, who had undergone periodontal surgery (eg, recession coverage using palatal subepithelial connective tissue grafts or crown lengthening) following signed informed consent and approval by the Ethics Committee of the University of Bern. Each donor tissue sample was minced into 1‐mm tissue explant pieces, which were then pipetted into a 25‐cm^3^ tissue culture flask and cultivated in Dulbecco's modified Eagle's medium (DMEM; Invitrogen, Basel, Switzerland) supplemented with 10% fetal calf serum (FCS; Invitrogen). Thus, each of the HPF and HGF strains originating from individual donors represents a mixture of fibroblasts that grew of multiple explants. Fibroblasts that had not undergone more than five passages were starved in 0.3% FCS/DMEM before treatment with HA.

Hyaluronan was kindly provided by Regedent AG (Zurich, Switzerland) in two formulations: (a) HA1 (hyaDENT), a native non‐cross‐linked HA with MW of 2500 kDa; and (b) HA2 (hyaDENT BG), a formulation containing complexes of butanediol diglycidyl ether (BDDE) cross‐linked 1000 kDa‐HA monomers and the above non‐cross‐linked form in a ratio of 8:1. According to the manufacturer, the sizes of the two HAs as well as the cross‐linking ratio are necessitated by the production process, potential of current technologies and application properties, eg, the applied cross‐linking ratio slows the resorption rate of HA2 while creating a gel‐type optimal for clinical application.

For RNA analyses, cells were plated at 3 × 10^4^ cells/cm^2^ on HA‐coated plates for 24 hour. For protein analyses, 6 hour after seeding at the same density, the adherent cells were starved in 0.3% FCS/DMEM for 18 hour and then treated with HA applied on the top for 30 minutes. For both types of analyses, HA was used at a final concentration of 4 mg/mL in 0.3%FCS/DMEM. This concentration was chosen based on (a) pilot experiments (data not shown) comparing 4 mg/mL‐diluted HAs with the undiluted commercial preparations and (b) the assumption that each of the HA preparations will be naturally diluted by patients' blood and saliva during the periodontal surgical procedure. In some cases, cells were treated with recombinant IL‐1α, IL‐1β or TNF‐α protein (PeproTech, London, UK) for 48 hours before RNA extraction.

### 2.2 Cell viability assay

Cell viability was assessed by the CellTiter‐Blue viability assay (Promega, Madison, WI, USA). After 24 hour of starvation, cells were plated in triplicate at 5 × 10^3^ cells/well on 96‐well plates coated with HA at the indicated concentrations (in the range of 0 and 4 mg/mL) prepared in 0.3%FCS/DMEM. Two hours post‐seeding, CellTiter‐Blue^®^ Reagent (20 μL/well) was added to the cells for 4 hour before recording fluorescence using a luminometer Infinite^®^ 200 (Tecan, Männedorf, Switzerland). Experimental values were normalized to the values of untreated cells (100% viability). Data represent means ± SD from three independent experiments performed with three different cell donors, each in triplicates.

### 2.3 Cell proliferation assay

Proliferation rates of HA‐treated HPF or HGF cells were determined using a 5‐bromo‐20‐deoxyuridine (BrdU) incorporation assay (Roche, Basel, Switzerland) as described.^27^, ^28^ After 24 hour of starvation, cells were plated in triplicate at 2 × 10^3^ cells/well on 96‐well plates coated with HA at a final concentration of 4 mg/mL in 0.3%FCS/DMEM. Cells were allowed to proliferate for 0, 24, 48, 72, and 96 hour before labeling with BrdU for 2 hour. BrdU incorporation into newly synthesized DNA was determined according to manufacturer's instructions. Experimental values were normalized to the values of untreated cells at the time point 0. Data represent means ± SD from three independent experiments performed with three different cell donors, each in triplicates.

### 2.4 Cell migration assay

Cell migration was assayed using transwell polycarbonate membrane inserts (Corning, Amsterdam, The Netherlands) as described.^27^ After 24 hour of starvation, 5 × 10^4^ cells were plated in the top insert chamber in serum‐free DMEM. The lower chamber was coated with HA at a final concentration of 4 mg/mL in 10% FCS/DMEM. Cells were allowed to migrate across the filter for 18 hour at 37°C before fixation and crystal violet staining. Images of duplicate inserts were acquired on an Olympus BX‐51 microscope. Migration was quantified by using the Fiji distribution of ImageJ as published before.^27^ Data represent means ± SD from three independent experiments performed with three different cell donors, each in triplicates.

### 2.5 qRT‐PCR

Quantitative RT‐PCR was used to investigate the expression of COL1A1, COL3A1, TGFB1, TGFB3, PDGFB, FGF2, EGF, IL1A, IL1B, TNF, MMP1, 2, 3, and 8 genes. Total RNA from HA‐ or growth factor‐treated cells was isolated using the RNeasy Mini Kit (Qiagen, Basel, Switzerland). RNA was reverse transcribed and relative transcripts for the above genes, normalized to GAPDH, were measured using FastStart Universal SYBR Green Master ROX (Roche) and the primer sequences listed in Table S1. Real‐time PCR was carried out in a 7500 Real‐Time PCR System (Applied Biosystems, Foster City, CA, USA). Data were analyzed using the efficiency ∆∆Ct method.^29^ All samples were run in duplicates. Data represent means ± SD from three independent experiments performed with three different cell donors.

### 2.6 Immunoblotting

Whole‐cell extracts from HA‐treated HPF and HGF cells were prepared by lysis in RIPA buffer as described.^30^ Lysates were run on 10% SDS‐PAGE, and transferred to Amersham^™^ Protran^®^ membrane (Sigma, Basel, Switzerland). Proteins of interest were visualized using anti‐phospho‐Akt, anti‐Akt, anti‐phospho‐Erk1/2, anti‐Erk, anti‐phospho‐p38, anti‐p38 (all from Cell Signaling Technology, Danvers, MA, USA), and anti‐vinculin (Sigma) antibodies followed by horseradish peroxidase‐conjugated secondary antibodies (MP Biomedicals, Santa Ana, CA, USA) for detection with the SuperSignal^™^ West Dura Substrate (ThermoFisher Scientific, Zug, Switzerland). Phospho‐Akt, phospho‐Erk1/2 or phospho‐p38 protein expression relative to the respective total protein control was quantified by densitometry using ImageQuant (Molecular Dynamics, Groningen, The Netherlands). Data represent means ± SD from three independent experiments performed with three different cell donors.

### 2.7 Statistical analysis

All grouped data are means ± SD. Differences between groups were assessed by one‐way analysis of variance (ANOVA) with Tukey's post hoc test using GraphPad InStat Software, version 3.05. Values of *P* < 0.05 were considered statistically significant.

## 3 RESULTS

### 3.1 The two HA preparations exert no negative effects on the viability of primary HPF and HGF cells

Coating of cell culture plates with HA1 resulted in the formation of a continuous uniform gel layer onto which both cell types, HPF and HGF, were able to adhere and assumed a fibroblast‐specific spindle‐shaped morphology that did not differ from the morphology of untreated cells seeded on non‐coated cell culture plastic (Figure S1). In contrast, coating with HA2 resulted in the formation of HA meshes, most likely due to HA2 being cross‐linked to BDDE. Thus, cells appeared to adhere solely on the cell culture plastic while HA2 was present in suspension (Figure S1). Due to the observed difference in the natural occurrence of the two HAs, a further distinction between HA applied as a coating or in suspension was not made and we referred to “HA treatment” throughout the study.

**Figure 1:**
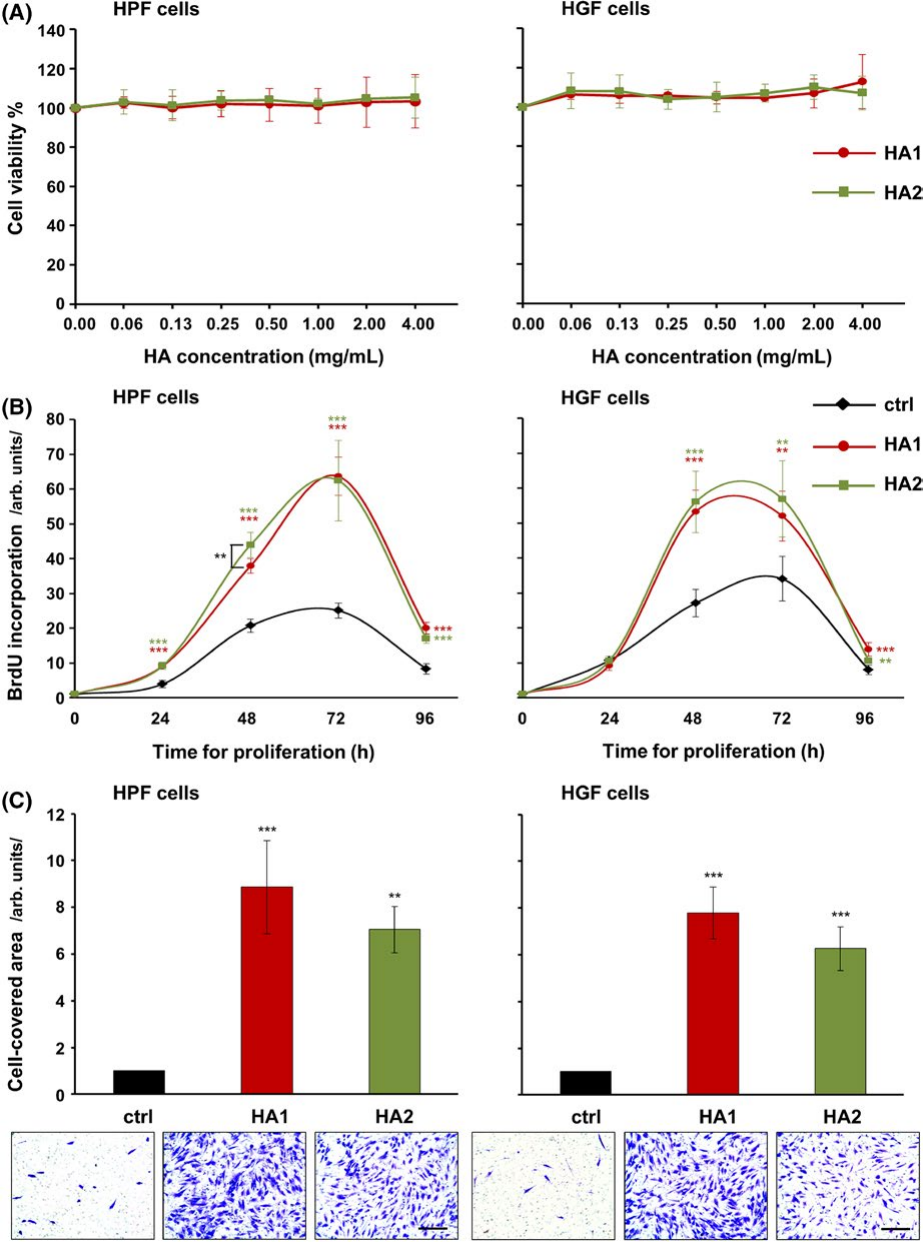
Effects of hyaluronan (HA) preparations on the metabolism and behavior of primary human palatal (HPF) and gingival (HGF) fibroblasts. A, The two HA preparations exert no negative effects on the viability of primary oral fibroblasts. Viability of HPF and HGF cells incubated with various concentrations (0‐4 mg/mL) of each of the two HA preparations was assessed by measuring the metabolic capacity using the CellTiter‐Blue^®^ Cell Viability Assay. Viable cells retain the ability to reduce the indicator dye resazurin into highly fluorescent resorufin. Experimental values were normalized to those of control cells (0 mg/mL HA; 100% viability). Data represent means ± SD from three independent experiments. B and C, The two HA preparations strongly increase the proliferative and migratory abilities of primary oral fibroblasts. B, Proliferation rates of HA‐treated HPF and HGF cells were assessed by BrdU incorporation into newly synthesized DNA immediately after plating (0 h) as well as at 24, 48, 72, and 96 h. Means ± SD from three independent experiments and significant differences to control (ctrl) cells at the time point 0, ***P < 0.001, **P < 0.01 are shown. C, Migration of HPF and HGF cells toward HA was evaluated by transwell migration assay using filters with 8 μm pore size. Cell migration was quantified by measuring the area on the lower side of the filter covered with cells. Means ± SD from three independent experiments and significant differences to control cells, ***P < 0.001, **P < 0.01 are shown. Representative images of the staining in each of the experimental groups are shown below the bar graph. Scale bar, 500 μm

We first compared the effects of the two HA preparations on HPF and HGF cell viability. Relative to control cells (0 mg/mL HA; 100% cell viability), fibroblasts exposed to HA1 or HA2 at various concentrations (0‐4 mg/mL) maintained a high level of viability (≥100%; Figure 1A).

### 3.2 The two HA preparations strongly increase the proliferative and migratory abilities of primary HPF and HGF cells

Next, we assessed the effects of the two HA preparations on the proliferation rates of HPF and HGF cells (Figure 1B). Compared to untreated control cells, HPF cells treated with either HA1 or HA2 showed a significant increase in BrdU uptake into newly synthesized DNA until they reached confluence 72 hours later (Figure 1B, left panel). In contrast, HGF cells exposed to each of the two HAs behaved like control cells in the first 24 hours (Figure 1B, right panel). Thereafter, they showed a significant increase in BrdU uptake until they reached confluence 48 hours later. Compared to HA1, the pro‐proliferative effect of HA2 appeared to be slightly more pronounced in both cell types, but a significant difference (*P* < 0.01) in the activity of the two HAs was only detected in HPF cells 48 hours post treatment (Figure 1B, left panel).

Next, we examined the migratory capacity of oral fibroblasts toward HA using a transwell assay. Similarly to the effect on cellular proliferation, each of the two HA preparations significantly induced fibroblast cell migration by 6‐9‐fold compared to control cells (*P* < 0.01; Figure 1C).

Taken together, these results showed a strong pro‐proliferative and pro‐migratory effect of HA on primary oral fibroblasts with no significant differences in the potency between the two HA formulations.

### 3.3 The two HA preparations trigger expression of COL3A1 and TGFB3 genes characterizing scarless wound healing

**Figure 2:**
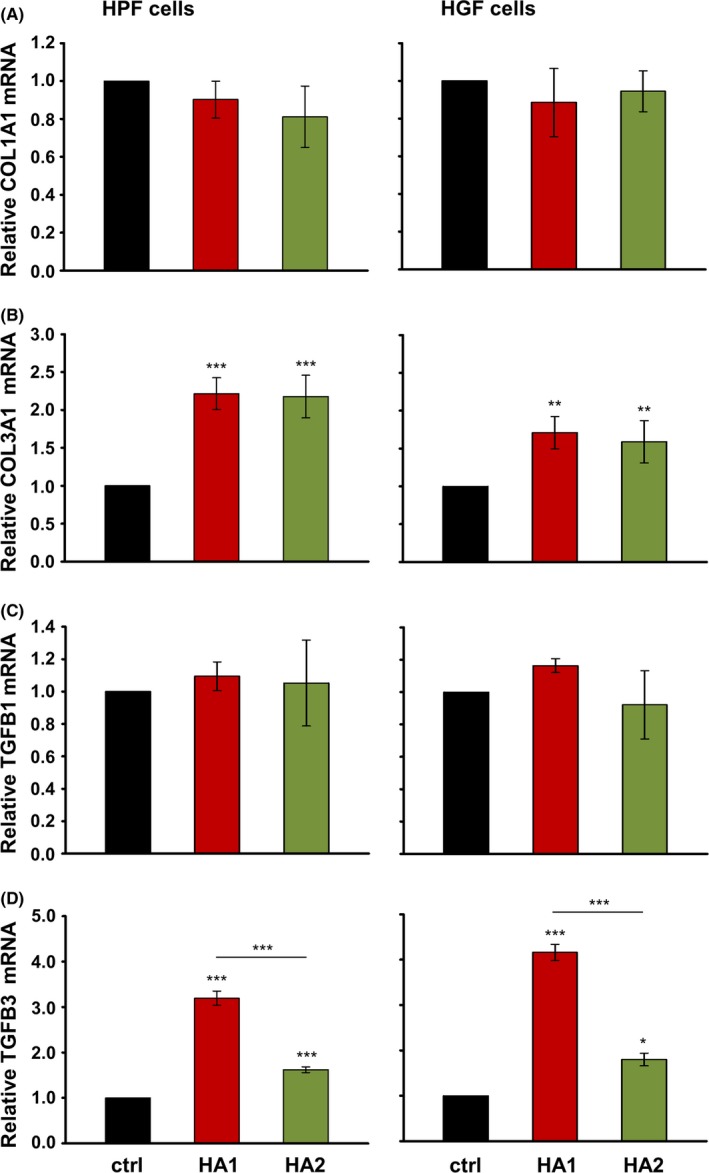
The two hyaluronan (HA) preparations trigger expression of COL3A1 and TGFB3 genes characterizing scarless wound healing. Effect of HA1 and HA2 on COL1A1 (A), COL3A1 (B), TGFB1 (C), and TGFB3 (D) mRNA levels in primary human palatal (HPF) and gingival (HGF) fibroblasts. Oral fibroblasts were treated with each of the two HA preparations for 24 h before total RNA was extracted and analyzed by qRT‐PCR. Values normalized to GAPDH are expressed relative to the values of untreated control (ctrl) cells. Data represent means ± SD from three independent experiments. Significant differences to the respective control unless otherwise indicated, ***P < 0.001, **P < 0.01, *P < 0.05

Invasion and proliferation of fibroblasts at the defect site precedes collagen deposition. Thus, we next studied the effect of the HA preparations on the expression of type I and type III collagens in the primary oral fibroblasts (Figure 2A and B). Accumulation of type I collagen is enhanced in scars and fibrosis, whereas type III collagen is abundant during fetal development and scarless fetal wound healing.^31^ Interestingly, compared to untreated control cells, either HA preparation upregulated the expression levels of COL3A1 in both HPFs and HGFs at 24 hour (Figure 2B), whereas no effect on COL1A1 mRNA levels was detected (Figure 2A).

We further determined the influence of the HA preparations on the expression of TGFB1 and TGFB3 (Figure 2C and D), two TGF‐β isoforms that play critical roles in wound healing by modulating ECM formation. Whereas TGF‐β1 is generally known as pro‐fibrotic, TGF‐β3 is the isoform that predominates in scarless fetal wound healing.^32^ Interestingly, both HAs caused a significant increase in TGFB3 mRNA levels compared to control fibroblasts (Figure 2D), whereas the expression of TGFB1 was unchanged (Figure 2C). Furthermore, in both cell types, HA1 appeared to be more potent (by 2‐fold, *P* < 0.001) than HA2 in inducing TGFB3 expression (Figure 2D).

These results indicated that both HAs favor the expression of genes characteristic of scarless vs pro‐fibrotic wound healing.

### 3.4 HA induces expression of genes encoding growth factors and cytokines involved in wound healing in primary HPF and HGF cells

**Figure 3:**
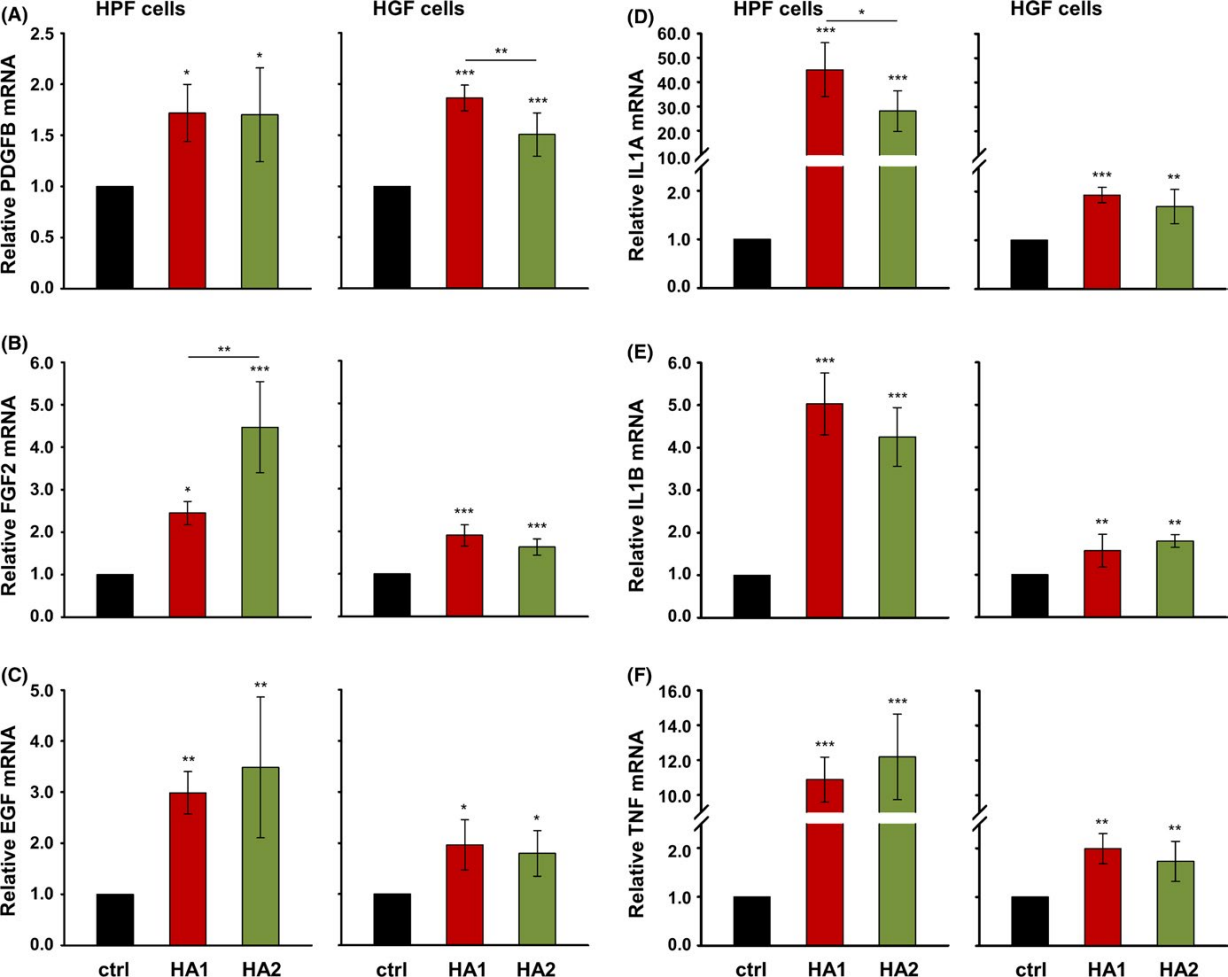
Hyaluronan (HA) induces expression of genes encoding growth factors and cytokines essential for the wound healing process in primary human palatal (HPF) and gingival (HGF) fibroblasts. Effect of HA1 and HA2 on PDGFB (A), FGF2 (B), EGF (C), IL1A (D), IL1B (E), and TNF (F) mRNA levels in HPF and HGF cells. Oral fibroblasts were treated with each of the two HA preparations for 24 h before total RNA was extracted and analyzed by qRT‐PCR. Values normalized to GAPDH are expressed relative to the values of untreated control (ctrl) cells. Data represent means ± SD from three independent experiments. Significant differences to the respective control unless otherwise indicated, ***P < 0.001, **P < 0.01, *P < 0.05

Growth factors such as PDGFB, FGF‐2 and EGF stimulate the proliferation and migration of human gingival fibroblasts in a dose‐dependent manner.^33^, ^34^ Thus, we investigated if the HA formulations might affect cellular proliferation and migration through influencing the expression of these growth factors. qRT‐PCR analyses revealed a significant induction of all three growth factors in both HPFs and HGFs exposed to each of the two HAs compared to basal expression levels in control cells (Figure 3A‐C). In isolated cases, one HA appeared to be more potent than the other, eg, HA1 caused a significantly higher induction of PDGFB mRNA in HGF cells (Figure 3A, right panel), whereas HA2 more strongly induced FGF2 expression in HPF cells (Figure 3B, left panel).

We next examined the effect of the two HAs on the expression of the pro‐inflammatory cytokine genes IL1A, IL1B and TNF (Figure 3D‐F). Similarly to the effect on growth factor expression, both HAs caused a significant upregulation of mRNA levels for each cytokine in both HPFs and HGFs. A slightly but significantly higher induction of IL1A expression was triggered by HA1 compared to HA2 in HPFs (Figure 3D, left panel). Interestingly, both HAs appeared to stimulate pro‐inflammatory cytokine expression more potently in HPF compared to HGF cells (Figure 3D‐F, compare left with right panels). The comparison between the two cell types was possible based on (a) identical basal expression levels for each of the three mRNAs encoding pro‐inflammatory cytokines in control HPFs and HGFs (Ct_ctrl _= 33 for IL1A; Ct_ctrl _= 30 for IL1B, and Ct_ctrl _= 31 for TNF) and (b) identical endogenous HA expression levels in culture supernatants of the two cell types (Figure S2).

In summary, the two HA preparations caused upregulation of wound healing‐related growth factors and cytokines in primary oral fibroblasts, with a more moderate effect on pro‐inflammatory cytokine expression in gingival compared to palatal fibroblasts. No clear trend for a difference in efficacy between the two HA formulations was observed.

### 3.5 HA induces cell type‐specific differences in the expression of MMP2 and MMP3 genes

**Figure 4:**
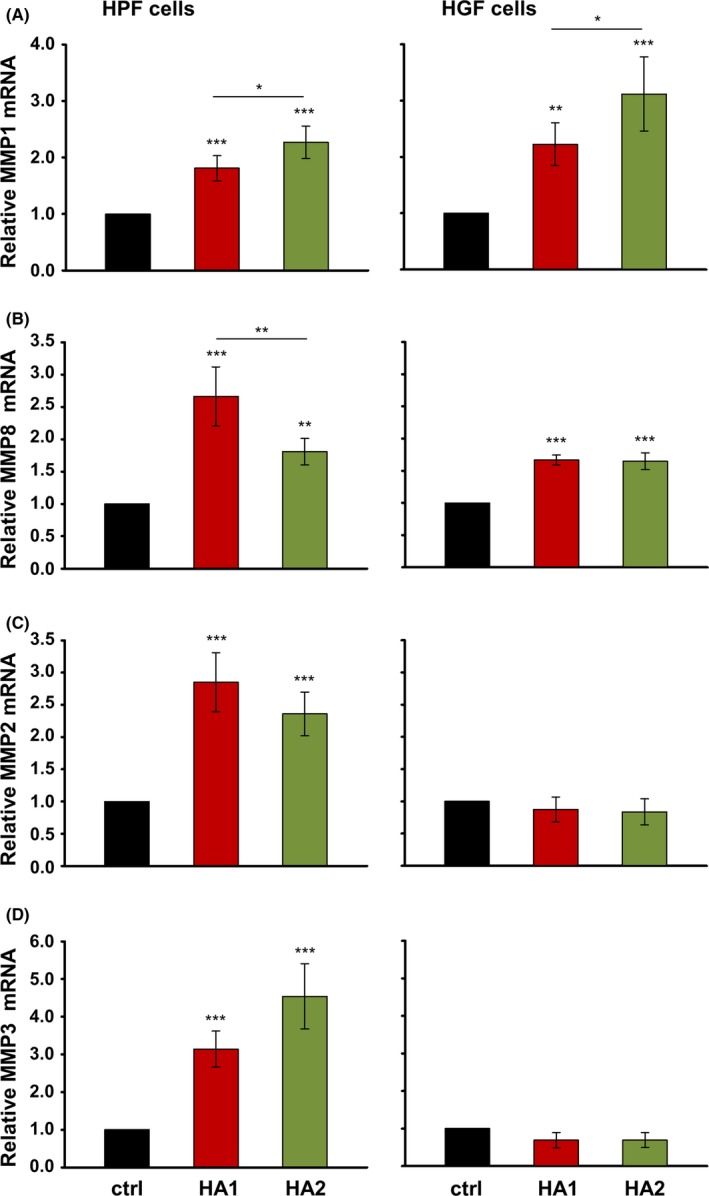
Hyaluronan (HA) induces cell type‐specific differences in the expression of MMP2 and MMP3 genes. Effect of HA1 and HA2 on MMP1 (A), MMP8 (B), MMP2 (C), and MMP3 (D) mRNA levels in primary human palatal (HPF) and gingival (HGF) fibroblasts. Oral fibroblasts were treated with each of the two HA preparations for 24 h before total RNA was extracted and analyzed by qRT‐PCR. Values normalized to GAPDH are expressed relative to the values of untreated control (ctrl) cells. Data represent means ± SD from three independent experiments. Significant differences to the respective control unless otherwise indicated, ***P < 0.001, **P < 0.01, *P < 0.05

During oral wound healing, the granulation tissue ECM is continuously remodeled by MMPs, most notably collagenases MMP‐1 and 8, major gelatinase MMP‐2, and stromelysin‐1 (MMP‐3).^35^ qRT‐PCR analyses revealed that treatment of both cell types with HAs resulted in an increase in MMP1 and MMP8 mRNA levels above those obtained in control cells (Figure 4A and B). Whereas HA2 appeared to be slightly more potent than HA1 for induction of MMP1 in both HPF and HGF cells (Figure 4A), HA1 caused a higher expression of MMP8 than HA2 in HPFs only (Figure 4B, left panel). Interestingly, we observed a significant induction of MMP2 mRNA levels in HA‐treated HPF but not HGF cells (Figure 4C), despite the facts that (a) MMP2 is constitutively expressed by mucosal fibroblasts^36^ and (b) basal expression levels of MMP2 were the same in control HPF and HGF cells, respectively (Ct_ctrl _= 20). Furthermore, at identical basal expression levels (Ct_ctrl _= 24) in control cells of both types, a 3‐5‐fold induction was evident for MMP3 upon HA treatment of HPF but not HGF cells (Figure 4D).

Thus, we observed cell type‐specific differences in the expression of two of the four MMPs tested upon HA treatment.

### 3.6 Pro‐inflammatory cytokines exhibit a stimulatory and dose‐dependent effect on MMP2 and MMP3 gene expression in primary HPF and HGF cells

**Figure 5:**
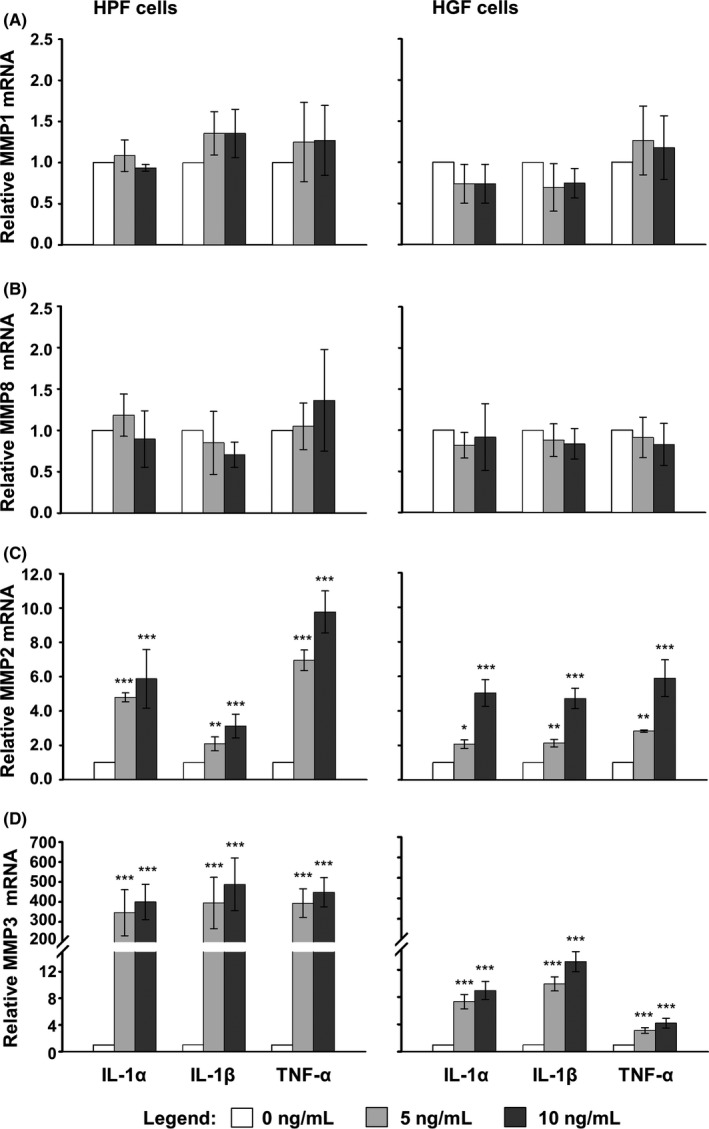
Pro‐inflammatory cytokines exhibit a stimulatory and dose‐dependent effect on MMP2 and MMP3 gene expression in primary human oral fibroblasts. Effect of recombinant IL‐1α, IL‐1β, andTNF‐α on the expression of MMP genes in primary human palatal (HPF) and gingival (HGF) fibroblasts. Cells were treated with increasing concentrations (0, 5, and 10 ng/mL) of each of the three pro‐inflammatory cytokines for 24 h before total RNA was extracted. Expression of MMP1 (A), MMP8 (B), MMP2 (C), and MMP3 (D) mRNAs was analyzed by qRT‐PCR. Values normalized to GAPDH are expressed relative to the values of untreated cells (0 ng/mL HA). Means ± SD from three independent experiments and significant differences to untreated cells, ***P < 0.001, **P < 0.01, *P < 0.05 are shown

The pro‐inflammatory cytokines IL‐1α, IL‐1β, and TNF‐α are known to play a major role in regulating MMP expression in different fibroblast cell lines and inflammatory diseases.^35^ To investigate the mechanism behind the differential induction of MMP2 and MMP3 by HA in HPF vs HGF cells, we treated oral fibroblasts with recombinant pro‐inflammatory cytokines and examined the effect on MMP expression. qRT‐PCR analyses revealed that increasing concentrations (5 and 10 ng/mL) of IL‐1α, IL‐1β, or TNF‐α applied to HPF or HGF cells had no effect on the expression of MMP1 and MMP8 mRNA levels (Figure 5A and B) but caused a significant and dose‐dependent increase in MMP2 and MMP3 mRNAs (Figure 5C and D) compared to their levels in untreated cells. This suggests that whereas HA might independently influence MMP1 and MMP8 gene expression (cf. Figure 4A, B), the effect of HA on MMP2 and MMP3 expression in HPF and HGF cells appears to occur indirectly and reflects the HA‐induced expression of pro‐inflammatory cytokines (cf. Figures 4C, D and 3D‐F).

### 3.7 The two HA preparations significantly enhance phosphorylation of Akt, Erk1/2 and p38 kinases in primary oral fibroblasts

**Figure 6:**
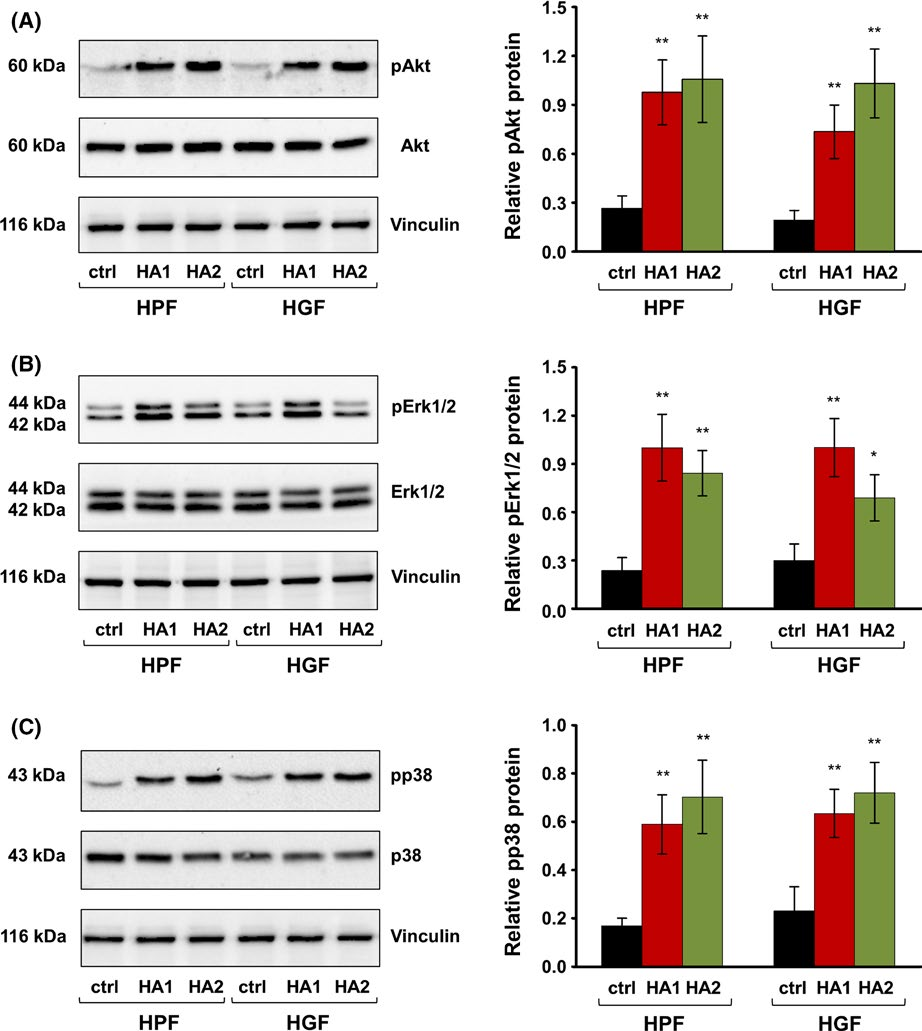
The two hyaluronan (HA) preparations significantly enhance phosphorylation of Akt, Erk1/2, and p38 kinases in primary human oral fibroblasts. Immunoblot analyses of phospho‐Akt (pAkt) (A), phospho‐Erk1/2 (pErk1/2) (B), and phospho‐p38 (pp38) (C) proteins in whole‐cell extracts from HA‐treated primary human palatal (HPF) and gingival (HGF) fibroblasts. Blots for total Akt, Erk1/2, and p38 proteins as well as the vinculin loading control are also shown. The bar charts represent densitometric quantifications of the immunoblots. pAkt, pErk1/2, and pp38 levels are normalized to the respective total proteins used as internal controls. Data represent means ± SD from three independent experiments. Significant differences to the respective control (ctrl) cells of each of the two cell types, **P < 0.01, *P < 0.05

To gain insights into the mechanisms whereby the two HAs exert their effects on gene expression in primary oral fibroblasts, we investigated the activation state of signaling kinases that might be triggered by HA. We focused on the activation of PI3‐kinase/Akt, Erk1/2, and p38, because HA has been reported to stimulate these pathways in other systems.^9^ Compared to the basal levels of phospho‐Akt, phospho‐Erk1/2, and phospho‐p38 detected in control cells, phosphorylation of each of the three kinases significantly increased in both HPFs and HGFs treated with either HA formulation for 30 minutes (Figure 6). In particular, the phosphorylation of Akt in HA‐treated cells was upregulated between 3.7 and 5.4‐fold compared to control cells (Figure 6A), whereas the phosphorylation of Erk1/2 and p38 increased by 2.3‐4.3‐ and 2.8‐4.2‐fold, respectively (Figure 6B and C).

These results indicated that each of the two HA formulations was able to trigger downstream signaling events through activation of Akt, Erk1/2, or p38 kinases in primary oral fibroblasts. No trend for a more pronounced effect of one or the other HA on the activation of the three signaling pathways was observed.

## 4 DISCUSSION

Palatal and gingival fibroblasts play an important role in oral wound healing. The use of bioactive substances that influence their behavior and thus support oral soft tissue wound healing/regeneration is of major clinical interest. Therefore, the aim of the present study was to investigate the specific role of two commercially available HA formulations in affecting oral fibroblast cell behavior, including proliferation, migration, and wound healing‐related gene expression. All these processes affect soft tissue wound healing/regeneration following reconstructive periodontal surgery. Our data demonstrate that both formulations of HA (a) are fully biocompatible and exert no negative effects on the viability of HPFs and HGFs; (b) are able to increase the proliferative and migratory abilities of both cell types; (c) trigger expression of COL3A1 and TGFB3 genes characterizing scarless fetal wound healing; (d) upregulate the expression of genes encoding the growth factors PDGFB, FGF‐2, and EGF, which are essential for the wound healing process; (e) induce pro‐inflammatory cytokine gene expression, thus potentially initiating a cellular inflammatory response; and (f) affect MMP gene expression either directly (MMP1 and 8) or indirectly (MMP2 and 3), potentially through induction of pro‐inflammatory cytokines, thus influencing ECM remodeling. Finally, (g) our data point on Akt, Erk1/2, and p38 as the signaling molecules by which the two HA preparations exert their effects on oral fibroblasts. Understanding the mechanisms whereby these HAs function may reveal how to intervene in the dynamic process of oral soft tissue regeneration with the aim to improve it.

The investigated HA preparations appear close to the physiological HA that is found in many biological fluids and solid tissues and is reported to possess an average MW of approximately 1000‐6000 kDa.^37^ Little if any HA below 1000 kDa is found in healthy solid tissues.^37^ On the other hand, human amniotic fluid contains HA with an average MW of approximately 330 kDa at 16 weeks gestation and is characterized with a change to a mixture of high and very low MW HA by 40 weeks gestation.^38^ The maintained high viability of HA‐treated fibroblasts in our study might be due to the physiochemical properties of high MW HA that is shown to exhibit an optimal viscoelasticity, prolonged dwell time, and extended biocompatibility.^39^ Favorable effects on viability of odontoblasts, fibroblasts, and periodontal ligament (PDL) cells have been reported previously with other HA formulations.^40^, ^41^ The cross‐linking to BDDE, which characterizes one of our HA formulations (HA2), did not seem to negatively influence cell viability as also shown by others.^42^

The effect of HA on cell proliferation in vitro is known to be closely related to its MW and concentration.^22^ Low MW HA was reported to increase cell proliferation in studies with rat and mouse mesenchymal stem cells,^43^, ^44^ rabbit bone marrow‐derived stem cells^45^ as well as in an organotypic keratinocyte‐fibroblast co‐culture model.^46^ In contrast, the reported effects of high MW HA on cellular proliferation are controversial.^22^ A significant stimulatory as well as an inhibitory effect has been seen dependent on the cellular context, HA concentrations, and method of delivery of HA to the cell culture. Here, we report a strong positive effect of both HA preparations on HPF and HGF cell proliferation. In addition to the pro‐proliferative effect, we observed a significantly enhanced migratory ability of oral fibroblasts toward HA. The HA‐induced cell motility might be due to enhanced Erk phosphorylation caused by the exogenous application of HA on oral fibroblasts that we have detected. Consistent with our findings, several groups have shown that HA activates Erk1,^47^ PI3K‐Akt,^48^ and p38^49^ signaling pathways that promote cell survival, motility, and proliferation. In accordance with those studies, the two HA formulations caused statistically significant induction of genes encoding pro‐proliferative and pro‐migratory growth factors such as PDGFB, FGF‐2, and EGF. Furthermore, once upregulated at the defect site, these factors might themselves stimulate synthesis of endogenous HA, as all three of them have been shown to enhance mRNA expression for specific HA synthases.^50^, ^51^ Future research is needed to identify the receptors that might be responsible for the observed effects of the two HA formulations on the behavior of oral fibroblasts. It is well known that native HA can bind to specific cell surface receptors such as CD44, RHAMM (receptor for hyaluronan‐mediated motility expressed protein), or LYVE‐1 (lymphatic vessel endothelial hyaluronan receptor‐1), thus inducing transduction of intracellular signals influencing cell survival, proliferation, and motility.^52^ Earlier research by Hirano et al^53^ have shown that periodontal tissues, in particular human gingival fibroblasts, human periodontal ligament fibroblasts, and human gingival epithelial cells all express CD44 protein and its mRNA. Furthermore, it has been shown that interaction between CD44 and other receptors such as epidermal growth factor receptor (EGFR) are essential for modulating HA‐dependent fibroblast proliferation.^54^

The high abundance of HA in amniotic fluid and embryonic tissues and its involvement in a mechanism that supports scarless repair in the fetus are well documented.^55^ Here, we report an upregulation of COL3A1 and TGFB3 gene expression in HA‐treated oral fibroblasts, suggesting that the HAs tested in our study might contribute to the generation of a fetal‐like cell environment that favors scarless healing. Thus, HA might appear particularly important in the early stages of wound repair when ECM deposition needs to be limited.

Exogenous high MW‐HA has been shown to be anti‐inflammatory, eg, in a T cell‐mediated liver injury model in mice^56^ or in fibroblast‐like synoviocytes in a human osteoarthritis model.^57^ On contrary, we observed an increased expression of pro‐inflammatory cytokines in oral fibroblasts treated with each of the two high MW‐HAs. Others have also shown that high MW‐HA stimulates production of IL‐1β, TNF‐α, and IL‐8 in human uterine fibroblasts.^12^ These contradictions might be related to the specific HA formulations as well as the cellular context used. However, as an inflammatory reaction is generally indispensable for the kick‐off of wound repair, the pro‐inflammatory effect of the HA preparations tested in the current study might appear clinically beneficial especially when the HAs are applied on non‐inflamed tissues during plastic‐esthetic periodontal surgery. Furthermore, it has been shown that the pro‐inflammatory cytokines IL‐1α, IL‐1β, and TNF‐α play a major role in regulating MMP expression in different cell types.^35^, ^58^, ^59^, ^60^, ^61^, ^62^, ^63^ The effects of the pro‐inflammatory cytokines on MMP expression appear to depend greatly on the cellular context. It has been reported that IL‐1α upregulates MMP1 and 3 mRNA and protein expression in dental pulp fibroblasts, thus inducing collagen degradation and pulp destruction.^60^ Furthermore, expression of MMP1, 2, and 3 in dental pulp fibroblasts was also stimulated by IL‐1β and TNF‐α.^59^ In contrast, Dasu and colleagues have shown that MMP‐1 mRNA expression was markedly increased with IL‐6 and TNF‐α treatment but remained unchanged with IL‐1β in the context of human dermal fibroblasts.^62^ In our system, treatment with each of the three pro‐inflammatory cytokines caused a significant increase in MMP2 and 3 transcripts but had no influence on MMP1 and 8, suggesting that the effect of HA on the expression of MMP2 and 3 in oral fibroblasts is indirect and reflects the HA‐induced expression of pro‐inflammatory cytokines. Both, prolonged inflammation as well as an excessive amount of MMPs acting for a long time on the healing tissue, are deleterious for the wound healing process. In this respect, the observed indirect effect on the expression of MMP2 and MMP3, as well as the moderate induction of pro‐inflammatory cytokines in HGFs, suggests that the investigated HA formulations are not likely to contribute to deleterious effects on the wound healing process.

Naturally, our in vitro experiments have certain limitations that can only be addressed by in vivo studies. Cross‐linking of bioactive substances aims to increase their rheological stability at the site of injury. However, the effect of cross‐linking might only be apparent in vivo as in vitro experiments are too short‐term to allow an effect on stability to become obvious. Furthermore, both HA preparations tested in our study are considered high MW. However, in vivo these HAs would undergo degradation to lower MW molecules following hyaluronidase activity during the postoperative period, eg, after root coverage, implant placement or sinus lift surgery, and will thus exert additional or even opposing effects on the regenerative process. Despite these clear limitations, the obtained in vitro results contribute to understanding the complexity of the wound healing process, as well as the clinical potential of the two hyaluronan preparations for oral soft tissue regeneration.

In conclusion, both HA formulations investigated in the current study exert diverse positive effects on human palatal and gingival fibroblasts, two cell types involved in soft tissue regeneration following periodontal reconstructive therapies that utilize palatal connective tissue or free gingival grafts. The observed pro‐proliferative, pro‐migratory and pro‐wound healing properties of the two HAs speak in favor of their clinical potential. However, animal research and/or clinical randomized controlled trials with long‐term follow‐up studies are needed to evaluate the clinical efficacy of the two HA preparations.

## CONFLICTS OF INTEREST

The authors report no conflicts of interest related to this study.

## References

[1] Embery G , Oliver WM , Stanbury JB , Purvis JA . The electrophoretic detection of acidic glycosaminoglycans in human gingival sulcus fluid. Arch Oral Biol. 1982;27:177‐179. 6805449 10.1016/0003-9969(82)90140-6

[2] Pogrel MA , Low MA , Stern R . Hyaluronan (hyaluronic acid) and its regulation in human saliva by hyaluronidase and its inhibitors. J Oral Sci. 2003;45:85‐91. 12930131 10.2334/josnusd.45.85

[3] Embery G , Waddington RJ , Hall RC , Last KS . Connective tissue elements as diagnostic aids in periodontology. Periodontol 2000. 2000;24:193‐214. 11276867 10.1034/j.1600-0757.2000.2240109.x

[4] Ohno S , Ijuin C , Doi T , Yoneno K , Tanne K . Expression and activity of hyaluronidase in human periodontal ligament fibroblasts. J Periodontol. 2002;73:1331‐1337. 12479638 10.1902/jop.2002.73.11.1331

[5] Bartold PM , Wiebkin OW , Thonard JC . Glycosaminoglycans of human gingival epithelium and connective tissue. Connect Tissue Res. 1981;9:99‐106. 6458453 10.3109/03008208109160247

[6] Bartold PM , Miki Y , McAllister B , Narayanan AS , Page RG . Glycosaminoglycans of human cementum. J Periodontal Res. 1988;23:13‐17. 2963899 10.1111/j.1600-0765.1988.tb01020.x

[7] Waddington RJ , Embery G , Last KS . Glycosaminoglycans of human alveolar bone. Arch Oral Biol. 1989;34:587‐589. 2512903 10.1016/0003-9969(89)90100-3

[8] Neuman MG , Nanau RM , Oruna‐Sanchez L , Coto G . Hyaluronic acid and wound healing. J Pharm Pharm Sci. 2015;18:53‐60. 25877441 10.18433/j3k89d

[9] David‐Raoudi M , Tranchepain F , Deschrevel B , et al. Differential effects of hyaluronan and its fragments on fibroblasts: relation to wound healing. Wound Repair Regen. 2008;16:274‐287. 18282267 10.1111/j.1524-475X.2007.00342.x

[10] Frost SJ , Weigel PH . Binding of hyaluronic acid to mammalian fibrinogens. Biochim Biophys Acta. 1990;1034:39‐45. 2328260 10.1016/0304-4165(90)90150-u

[11] Noble PW . Hyaluronan and its catabolic products in tissue injury and repair. Matrix Biol. 2002;21:25‐29. 11827789 10.1016/s0945-053x(01)00184-6

[12] Kobayashi H , Terao T . Hyaluronic acid‐specific regulation of cytokines by human uterine fibroblasts. Am J Physiol. 1997;273:C1151‐C1159. 9357758 10.1152/ajpcell.1997.273.4.C1151

[13] Slevin M , Kumar S , Gaffney J . Angiogenic oligosaccharides of hyaluronan induce multiple signaling pathways affecting vascular endothelial cell mitogenic and wound healing responses. J Biol Chem. 2002;277:41046‐41059. 12194965 10.1074/jbc.M109443200

[14] Larjava H , Heino J , Kähäri V‐M , Krusius T , Vuorio E . Characterization of one phenotype of human periodontal granulation‐tissue fibroblasts. J Dent Res. 1989;68:20‐25. 2910955 10.1177/00220345890680010301

[15] Schultz GS , Wysocki A . Interactions between extracellular matrix and growth factors in wound healing. Wound Repair Regen. 2009;17:153‐162. 19320882 10.1111/j.1524-475X.2009.00466.x

[16] Gill SE , Parks WC . Metalloproteinases and their inhibitors: regulators of wound healing. Int J Biochem Cell Biol. 2008;40:1334‐1347. 18083622 10.1016/j.biocel.2007.10.024 PMC2746915

[17] Werner S , Grose R . Regulation of wound healing by growth factors and cytokines. Physiol Rev. 2003;83:835‐870. 12843410 10.1152/physrev.2003.83.3.835

[18] Ignotz RA , Massagué J . Transforming growth factor‐beta stimulates the expression of fibronectin and collagen and their incorporation into the extracellular matrix. J Biol Chem. 1986;261:4337‐4345. 3456347

[19] Xie J , Bian H , Qi S , et al. Effects of basic fibroblast growth factor on the expression of extracellular matrix and matrix metalloproteinase‐1 in wound healing. Clin Exp Dermatol. 2008;33:176‐182. 18257838 10.1111/j.1365-2230.2007.02573.x

[20] Chen L , Tredget EE , Wu PYG , Wu Y . Paracrine factors of mesenchymal stem cells recruit macrophages and endothelial lineage cells and enhance wound healing. PLoS One. 2008;3:e1886. 18382669 10.1371/journal.pone.0001886 PMC2270908

[21] Bertl K , Bruckmann C , Isberg P‐E , Klinge B , Gotfredsen K , Stavropoulos A . Hyaluronan in non‐surgical and surgical periodontal therapy: a systematic review. J Clin Periodontol. 2015;42:236‐246. 25640222 10.1111/jcpe.12371

[22] Zhao N , Wang X , Qin L , et al. Effect of hyaluronic acid in bone formation and its applications in dentistry. J Biomed Mater Res A. 2016;104:1560‐1569. 27007721 10.1002/jbm.a.35681

[23] Ballini A , Cantore S , Capodiferro S , Grassi FR . Esterified hyaluronic acid and autologous bone in the surgical correction of the infra‐bone defects. Int J Med Sci. 2009;6:65‐71. 19277251 10.7150/ijms.6.65 PMC2653787

[24] Sehdev B , Bhongade ML , Ganji KK . Evaluation of effectiveness of hyaluronic acid in combination with bioresorbable membrane (poly lactic acid‐poly glycolic acid) for the treatment of infrabony defects in humans: a clinical and radiographic study. J Indian Soc Periodontol. 2016;20:50‐56. 27041838 10.4103/0972-124X.170809 PMC4795135

[25] Kumar R , Srinivas M , Pai J , Suragimath G , Prasad K , Polepalle T . Efficacy of hyaluronic acid (hyaluronan) in root coverage procedures as an adjunct to coronally advanced flap in Millers Class I recession: a clinical study. J Indian Soc Periodontol. 2014;18:746‐750. 25624632 10.4103/0972-124X.147411 PMC4296460

[26] Pilloni A , Schmidlin PR , Sahrmann P , Sculean A , Rojas MA . Effectiveness of adjunctive hyaluronic acid application in coronally advanced flap in Miller class I single gingival recession sites: a randomized controlled clinical trial. Clin Oral Investig. 2018; 10.1007/s00784-018-2537-4. [Epub ahead of print] 29961138

[27] Gurbuz I , Ferralli J , Roloff T , Chiquet‐Ehrismann R , Asparuhova MB . SAP domain‐dependent Mkl1 signaling stimulates proliferation and cell migration by induction of a distinct gene set indicative of poor prognosis in breast cancer patients. Mol Cancer. 2014;13:22. 24495796 10.1186/1476-4598-13-22 PMC3933235

[28] Asparuhova MB , Caballé‐Serrano J , Buser D , Chappuis V . Bone‐conditioned medium contributes to initiation and progression of osteogenesis by exhibiting synergistic TGF‐β1/BMP‐2 activity. Int J Oral Sci. 2018;10:20. 29895828 10.1038/s41368-018-0021-2 PMC5997631

[29] Livak KJ , Schmittgen TD . Analysis of relative gene expression data using real‐time quantitative PCR and the 2(‐Delta Delta C(T)) method. Methods. 2001;25:402‐408. 11846609 10.1006/meth.2001.1262

[30] Asparuhova MB , Ferralli J , Chiquet M , Chiquet‐Ehrismann R . The transcriptional regulator megakaryoblastic leukemia‐1 mediates serum response factor‐independent activation of tenascin‐C transcription by mechanical stress. FASEB J. 2011;25:3477‐3488. 21705668 10.1096/fj.11-187310

[31] Cuttle L , Nataatmadja M , Fraser JF , Kempf M , Kimble RM , Hayes MT . Collagen in the scarless fetal skin wound: detection with Picrosirius‐polarization. Wound Repair Regen. 2005;13:198‐204. 15828945 10.1111/j.1067-1927.2005.130211.x

[32] Lichtman MK , Otero‐Vinas M , Falanga V . Transforming growth factor beta (TGF‐β) isoforms in wound healing and fibrosis. Wound Repair Regen. 2016;24:215‐222. 26704519 10.1111/wrr.12398

[33] Mumford JH , Carnes DL , Cochran DL , Oates TW . The effects of platelet‐derived growth factor‐BB on periodontal cells in an in vitro wound model. J Periodontol. 2001;72:331‐340. 11327060 10.1902/jop.2001.72.3.331

[34] Fujisawa K , Miyamoto Y , Nagayama M . Basic fibroblast growth factor and epidermal growth factor reverse impaired ulcer healing of the rabbit oral mucosa. J Oral Pathol Med. 2003;32:358‐366. 12787043 10.1034/j.1600-0714.2003.t01-1-00111.x

[35] Lindner D , Zietsch C , Becher PM , et al. Differential expression of matrix metalloproteases in human fibroblasts with different origins. Biochem Res Int. 2012;2012:875742. 22500233 10.1155/2012/875742 PMC3303709

[36] Salo T , Mäkelä M , Kylmäniemi M , Autio‐Harmainen H , Larjava H . Expression of matrix metalloproteinase‐2 and ‐9 during early human wound healing. Lab Invest. 1994;70:176‐182. 8139259

[37] Cowman MK , Lee H‐G , Schwertfeger KL , McCarthy JB , Turley EA . The content and size of hyaluronan in biological fluids and tissues. Front Immunol. 2015;6:261. 26082778 10.3389/fimmu.2015.00261 PMC4451640

[38] Dahl LB , Dahl IMS , Børresen A‐L . The molecular weight of sodium hyaluronate in amniotic fluid. Biochem Med Metab Biol. 1986;35:219‐226. 3707753 10.1016/0885-4505(86)90077-0

[39] Guidolin D , Franceschi F . Viscosupplementation with high molecular weight native hyaluronan. Focus on a 1500‐2000 KDa fraction (Hyalubrix^®^). Eur Rev Med Pharmacol Sci. 2014;18:3326‐3338. 25487947

[40] Bogović A , Nižetić J , Galić N , Zelježić D , Micek V , Mladinić M . The effects of hyaluronic acid, calcium hydroxide, and dentin adhesive on rat odontoblasts and fibroblast. Arh Hig Rada Toksikol. 2011;62:155‐161. 21705303 10.2478/10004-1254-62-2011-2076

[41] Akizuki T , Oda S , Komaki M , et al. Application of periodontal ligament cell sheet for periodontal regeneration: a pilot study in beagle dogs. J Periodontal Res. 2005;40:245‐251. 15853971 10.1111/j.1600-0765.2005.00799.x

[42] Nishi C , Nakajima N , Ikada Y . In vitro evaluation of cytotoxicity of diepoxy compounds used for biomaterial modification. J Biomed Mater Res. 1995;29:829‐834. 7593021 10.1002/jbm.820290707

[43] Pilloni A , Bernard GW . The effect of hyaluronan on mouse intramembranous osteogenesis in vitro. Cell Tissue Res. 1998;294:323‐333. 9799448 10.1007/s004410051182

[44] Huang L , Cheng YY , Koo PL , et al. The effect of hyaluronan on osteoblast proliferation and differentiation in rat calvarial‐derived cell cultures. J Biomed Mater Res A. 2003;66A:880‐884. 10.1002/jbm.a.10535 12926041

[45] Zhao N , Wang X , Qin L , Guo Z , Li D . Effect of molecular weight and concentration of hyaluronan on cell proliferation and osteogenic differentiation in vitro. Biochem Biophys Res Commun. 2015;465:569‐574. 26284973 10.1016/j.bbrc.2015.08.061

[46] Gu H , Huang L , Wong Y‐P , Burd A . HA modulation of epidermal morphogenesis in an organotypic keratinocyte‐fibroblast co‐culture model. Exp Dermatol. 2010;19:e336‐e339. 20500771 10.1111/j.1600-0625.2009.01052.x

[47] Zhang S , Chang MCY , Zylka D , Turley S , Harrison R , Turley EA . The hyaluronan receptor RHAMM regulates extracellular‐regulated kinase. J Biol Chem. 1998;273:11342‐11348. 9556628 10.1074/jbc.273.18.11342

[48] Sohara Y , Ishiguro N , Machida K , et al. Hyaluronan activates cell motility of v‐Src‐transformed cells via Ras‐mitogen‐activated protein kinase and phosphoinositide 3‐kinase‐Akt in a tumor‐specific manner. Mol Biol Cell. 2001;12:1859‐1868. 11408591 10.1091/mbc.12.6.1859 PMC37347

[49] Ohno S , Im H‐J , Knudson CB , Knudson W . Hyaluronan oligosaccharides induce matrix metalloproteinase 13 via transcriptional activation of NFkappaB and p38 MAP kinase in articular chondrocytes. J Biol Chem. 2006;281:17952‐17960. 16648633 10.1074/jbc.M602750200 PMC3139229

[50] Pienimäki J‐P , Rilla K , Fülöp C , et al. Epidermal growth factor activates hyaluronan synthase 2 in epidermal keratinocytes and increases pericellular and intracellular hyaluronan. J Biol Chem. 2001;276:20428‐20435. 11262389 10.1074/jbc.M007601200

[51] Li L , Asteriou T , Bernert B , Heldin C‐H , Heldin P . Growth factor regulation of hyaluronan synthesis and degradation in human dermal fibroblasts: importance of hyaluronan for the mitogenic response of PDGF‐BB. Biochem J. 2007;404:327‐336. 17324121 10.1042/BJ20061757 PMC1868797

[52] Turley EA , Noble PW , Bourguignon LYW . Signaling properties of hyaluronan receptors. J Biol Chem. 2002;277:4589‐4592. 11717317 10.1074/jbc.R100038200

[53] Hirano F , Hirano H , Hino E , et al. CD44 isoform expression in periodontal tissues: cell‐type specific regulation of alternative splicing. J Periodontal Res. 1997;32:634‐645. 9409458 10.1111/j.1600-0765.1997.tb00573.x

[54] Meran S , Luo DD , Simpson R , et al. Hyaluronan facilitates transforming growth factor‐β1‐dependent proliferation via CD44 and epidermal growth factor receptor interaction. J Biol Chem. 2011;286:17618‐17630. 21454519 10.1074/jbc.M111.226563 PMC3093837

[55] Longaker MT , Chiu ES , Adzick NS , Stern M , Harrison MR , Stern R . Studies in fetal wound healing. V. A prolonged presence of hyaluronic acid characterizes fetal wound fluid. Ann Surg. 1991;213:292‐296. 2009010 10.1097/00000658-199104000-00003 PMC1358347

[56] Nakamura K , Yokohama S , Yoneda M , et al. High, but not low, molecular weight hyaluronan prevents T‐cell‐mediated liver injury by reducing proinflammatory cytokines in mice. J Gastroenterol. 2004;39:346‐354. 15168246 10.1007/s00535-003-1301-x

[57] Wang CT , Lin YT , Chiang BL , Lin YH , Hou SM . High molecular weight hyaluronic acid down‐regulates the gene expression of osteoarthritis‐associated cytokines and enzymes in fibroblast‐like synoviocytes from patients with early osteoarthritis. Osteoarthritis Cartilage. 2006;14:1237‐1247. 16806998 10.1016/j.joca.2006.05.009

[58] Dayer JM , Beutler B , Cerami A . Cachectin/tumor necrosis factor stimulates collagenase and prostaglandin E2 production by human synovial cells and dermal fibroblasts. J Exp Med. 1985;162:2163‐2168. 2999289 10.1084/jem.162.6.2163 PMC2187983

[59] Wisithphrom K , Windsor LJ . The effects of tumor necrosis factor‐alpha, interleukin‐1beta, interleukin‐6, and transforming growth factor‐beta1 on pulp fibroblast mediated collagen degradation. J Endod. 2006;32:853‐861. 16934628 10.1016/j.joen.2006.03.017

[60] Wisithphrom K , Murray PE , Windsor LJ . Interleukin‐1 alpha alters the expression of matrix metalloproteinases and collagen degradation by pulp fibroblasts. J Endod. 2006;32:186‐192. 16500223 10.1016/j.joen.2005.10.055

[61] Noh EM , Kim JS , Hur H , et al. Cordycepin inhibits IL‐1β‐induced MMP‐1 and MMP‐3 expression in rheumatoid arthritis synovial fibroblasts. Rheumatology. 2009;48:45‐48. 19056796 10.1093/rheumatology/ken417

[62] Dasu MRK , Barrow RE , Spies M , Herndon DN . Matrix metalloproteinase expression in cytokine stimulated human dermal fibroblasts. Burns. 2003;29:527‐531. 12927975 10.1016/s0305-4179(03)00154-2

[63] Kunisch E , Kinne RW , Alsalameh RJ , Alsalameh S . Pro‐inflammatory IL‐1beta and/or TNF‐alpha up‐regulate matrix metalloproteases‐1 and ‐3 mRNA in chondrocyte subpopulations potentially pathogenic in osteoarthritis: in situ hybridization studies on a single cell level. Int J Rheum Dis. 2016;19:557‐566. 25291965 10.1111/1756-185X.12431

