# Supplementary material for: Activity of two hyaluronan preparations on primary human oral fibroblasts
Source: J Periodontal Res. 2018 Sep 27;54(1):33–45. doi: 10.1111/jre.12602 (PMC6586051; doi:10.1111/jre.12602)
Supplement: Supplementary file 1 [file JRE-54-33-s001.pdf]

Supporting Material for:

Activity of two hyaluronan preparations on primary human oral fibroblasts

Maria B. Asparuhova[1, 2]* | Deniz Kiryak[1, 2] | Meizi Eliezer[2] | Deyan Mihov[3] | Anton Sculean[2]

[1]Laboratory of Oral Cell Biology, School of Dental Medicine, University of Bern, Bern, Switzerland

[2]Department of Periodontology, School of Dental Medicine, University of Bern, Bern, Switzerland

[3]Biozentrum, University of Basel, Basel, Switzerland

Content:

Table S1. Primer sequences.

Figure S1. Morphology of HA–treated HPF and HGF cells.

Figure S2. Endogenous expression levels of HA in the HPF and HGF cell strains used in the study.

Table S1. Primer Sequences.

Gene symbol	Gene bank accession number	Primer pair (fwd/rev)	Amplicon size (bp)
COL1A1	NM_000088.3	5'-GAAGGGACACAGAGGTTTCAG-3'<br>5'-TAGCACCATCATTTCCACGA-3'	190
COL3A1	NM_000090.3	5'-AAGGAAATGATGGTGCTCCTG-3'<br>5'-AGCCTTGTAATCCTTGTGGAC-3'	176
TGFB1	NM_000660.6	5'-AACCCACAACGAAATCTATGAC-3'<br>5'-GGAATTGTTGCTGTATTTCTGG-3'	180
TGFB3	NM_001329938.1	5'-GAGCTCTTCCAGATCCTTCG-3'<br>5'-TTTCTAGACCTAAGTTGGACTCTC-3'	162
PDGFB	NM_002608.3	5'-ATCACCATGCAGATTATGCG-3'<br>5'-GCTCTATCTTTCTTTGGTCTGC-3'	219
FGF2	NM_002006.4	5'-ACATCAAGCTACAACTTCAAGC-3'<br>5'-CCGTAACACATTTAGAAGCCAG-3'	119
EGF	NM_001178130.2	5'-AAGATATACTTTGCCCATACAGCC-3'<br>5'-AGAGATTTCCCTCTGTCTGTCC-3'	160
IL1A	NM_000575.4	5'-GCATGGATCAATCTGTGTCTC-3'<br>5'-GGCTTGATGATTTCTTCCTCTG-3'	192
IL1B	NM_000576.2	5'-AACAGATGAAGTGCTCCTTCC-3'<br>5'-AAGGTGCTCAGGTCATTCTC-3'	189
TNF	NM_000594.3	5'-TCTTCTCCTTCCTGATCGTG-3'<br>5'-GAGGGTTTGCTACAACATGG-3'	185
MMP1	NM_001145938.1	5'-TAAAGACAGATTCTACATGCGCAC-3'<br>5'-AACAGCCCAGTACTTATTCCCT-3'	162
MMP8	NM_001304441.1	5'-TCCAGCAAGAACATTTCTTCC-3'<br>5'-CAGCCATATCTACAGTTAAGCC-3'	120
MMP2	NM_001127891.2	5′-GACCAGAATACCATCGAGACCA-3′<br>5′-GTGTAGCCAATGATCCTGTATGTG-3′	127
MMP3	NM_002422.4	5'-TTTGCAGTTAGAGAACATGGAG-3'<br>5'-ACGAGAAATAAATTGGTCCCTG-3'	157
GAPDH	NM_001256799.2	5′-ATCAAGAAGGTGGTGAAGCAG-3′<br>5′-TCGTTGTCATACCAGGAAATGAG-3′	178

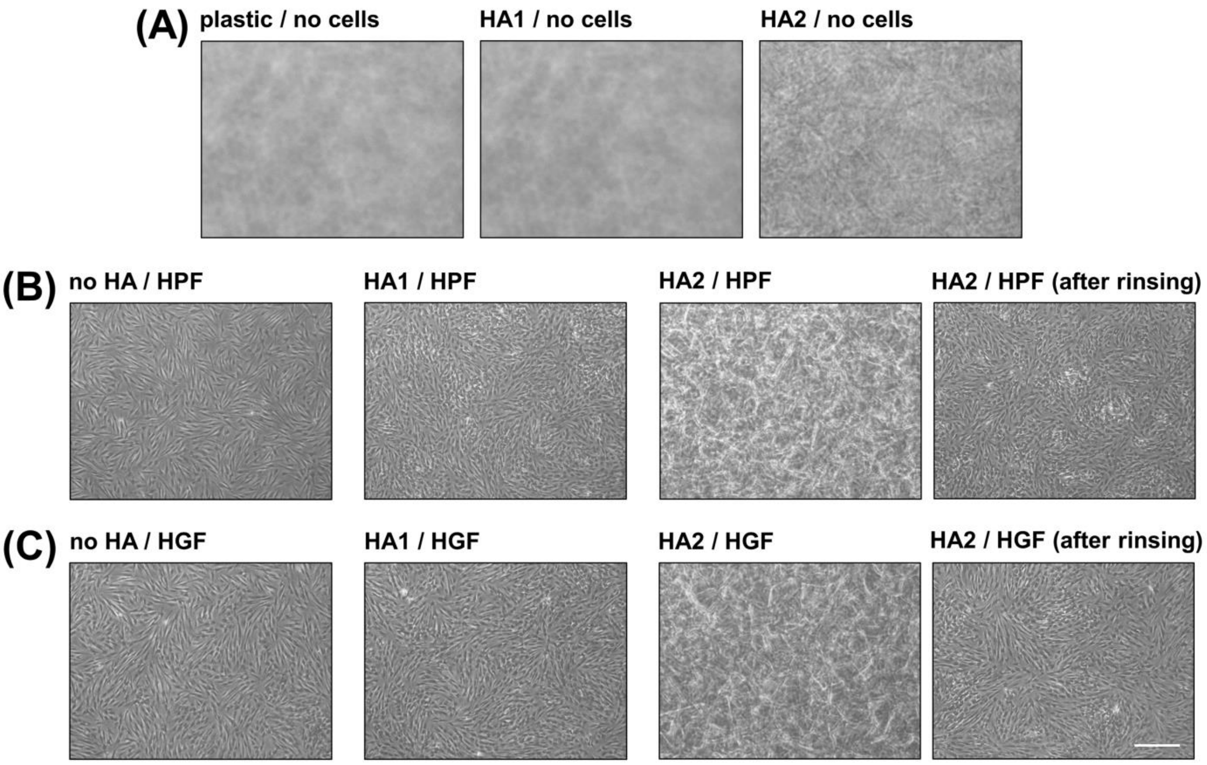

Figure S1. Morphology of HA–treated HPF and HGF cells. Coating of cell culture plates with HA1 resulted in the formation of a continuous uniform gel layer (A, middle panel), whereas coating with HA2 resulted in the formation of HA meshes (A, right panel). An image of an empty well with no coating is also presented (A, left panel). Both HAs were used at a final concentration of 4 mg/ml in 0.3% FCS/DMEM and coating was performed for 30 min. (B and C) Images of HPF (B) and HGF (C) cells seeded on uncoated (left panels, no HA), HA1- or HA2-coated (middle panels) wells. Cells were plated at 3 x 10^4 cells/cm^2 for 24 h before images were taken on a Leica DM IL LED microscope equipped with Leica DFC420 C camera. Both cell types, HPF and HGF, were able to adhere on HA1-coated plates and assumed a fibroblast-specific spindle-shaped morphology that did not differ from the morphology of untreated cells seeded on non-coated cell culture plastic (B and C, compare left with middle panels). In contrast, on HA2-coated plates, cells appeared to adhere solely on the cell culture plastic while HA2 was present in suspension. Images of adherent HGF cells after rinsing of the plate with PBS are shown (B and C, right panels). Bar, 500 µm.

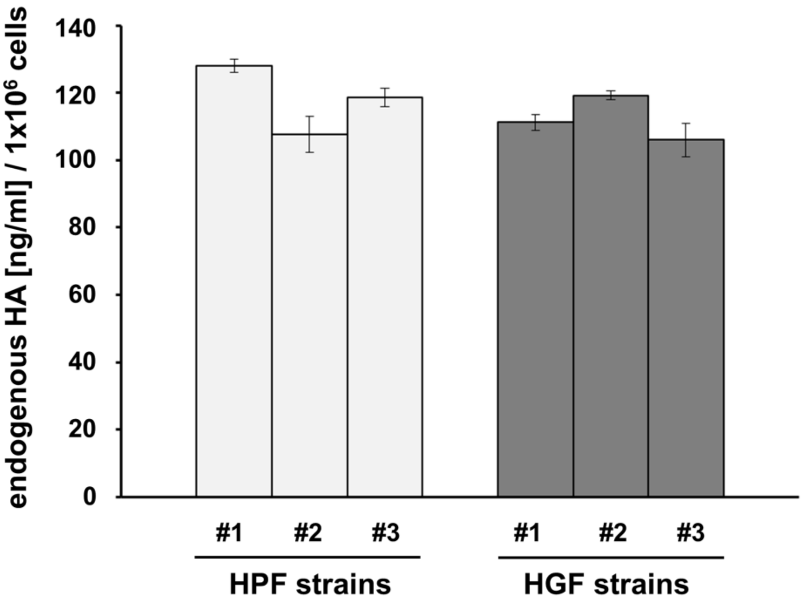

Figure S2. Endogenous expression levels of HA in the HPF and HGF cell strains used in the study. Release of HA in cell culture supernatants of HPF and HGF strains originating from three separate donors each was quantified using Quantikine® ELISA (R&D Systems, Zug, Switzerland) according to the manufacturer's procedure. After 24 h of starvation, cells were plated at 1 x 10^6 cells/well on 6-well plates in 0.3% FCS/DMEM for 24 h before cell culture supernatants were collected for the analysis. All samples were run in duplicates. Cell number estimated at the time of cell culture supernatant harvest was used for normalization. Data represent means ± SD from two independent experiments performed with each of the three cell donors.
